# Employment Opportunities and Experiences among Recent Master’s-Level Global Health Graduates

**DOI:** 10.5334/aogh.305

**Published:** 2019-03-13

**Authors:** William Cherniak, Elahe Nezami, Quentin Eichbaum, Jessica Evert, Ashti Doobay-Persaud, Sharon Rudy, Ginny DeFrank, Tom Hall, Adam Hoverman

**Affiliations:** 1 University of Toronto, Department of Family and Community Medicine, Division of Emergency Medicine – The Markham-Stouffville Hospital, CA; 2 Bridge to Health Medical and Dental, Toronto, CA; 3 University of Southern California, Keck School of Medicine, US; 4 Vanderbilt University, US; 5 Child Family Health International, University of California, San Francisco, CA, US; 6 Northwestern University, US; 7 Public Health Institute, Global Health Fellows Program-II, US; 8 University of California, San Francisco, US; 9 Oregon Health and Sciences University-Portland State University School of Public Health, Portland, OR, US

## Abstract

**Objectives:**

To examine the job search, employment experiences, and job availability of recent global health-focused master’s level graduates.

**Methods:**

An online survey was conducted from October to December 2016 based out of Washington, DC. The study sample includes students graduating with master’s degrees in global health, public health with a global health concentration or global medicine from eight U.S. universities.

**Results:**

Out of 256 potential respondents, 152 (59%) completed the survey, with 102/152 (67%) employed. Of unemployed graduates, 38% were currently in another educational training program. Out of 91 employed respondents, 62 (68%) reported they had limitations or gaps in their academic training. The average salary of those employed was between $40,000 and $59,000 annually. The majority of respondents reported they currently work in North America (83.5%.); however, only 31% reported the desire to work in North America following graduation.

**Conclusions:**

Discrepancies exist between graduates’ expectations of employment in global public health and the eventual job market. Communication between universities, students and employers may assist in curriculum development and job satisfaction for the global public health workforce.

## Introduction

In 1984, Baker et al. described career opportunities within international health and global health for graduates from public health training programs [1]. Over 35 years later, there are renewed concerns about mismatches between graduates and employment [2]. Specifically, since Baker’s study, the global health workforce has changed considerably, with health systems increasingly relying upon local and nationally trained staff and management. As such, the impetus has increased for universities in high-income countries (HICs) to develop programs that provide graduates the diversification of skills to fit current global health demands [1,3]. A 2016 survey of global health employers demonstrated the need for skills in program management, monitoring and evaluation (M&E), communications, strategy/project design, collaboration and teamwork [4]. Over time, two types of global health professionals have emerged: one focused on clinical care (e.g., health workers) and a second program-oriented (e.g., non-clinical systems-level workers) [5]. Another approach that has educational and competency implications is sorting positions in three ways: (1) direct service providers, (2) researchers and (3) implementers (reference, S. Rudy, GHFP-II private correspondence).

Existing data suggests 50% of job postings require knowledge and skills typically acquired in schools of public health, 51% require at least a master’s-level qualification or doctoral degree and a majority require five to seven years of international experience for internationally focused positions [6]. A trend toward domestic employment for trainees who have previously gained experience abroad has also been reported [7].

Expansion of global health education programs is well documented and presumed to be the result of a rapid increase in students interested in social accountability, health equity and health advocacy, rather than a reflection of an increase in employer demand [8,9,10,11]. In light of increasing numbers of graduates and shifting priorities around roles, the World Health Organization (WHO) recently called for improved global monitoring and accountability on international human resources for health goals [12].

Significant intra-national health disparities exist in many countries and are highlighted as impetus for a global health workforce with domestically focused expertise [13,14,15]. Yet, despite uncertain job prospects and reduced funding for international activities, students are frequently drawn to international global health programs by the glamor of working in far-away low- and middle-income country settings (LMICs), as well as the desire to do meaningful work [2,4,16,17].

The interdependence of both the global health training environments and the global health workforce has been formally described as the intersection of education and health systems [18]. This survey sought to provide a snapshot of the experience and outcomes of the job searches of recent graduates of master’s-level programs in global health, specifically with the hypothesis that the recent boom in training programs and evolution of global health jobs has created a mismatch between global health training, graduate aspirations and job availability.

## Methods

The Institutional Review Board at the Public Health Institute (Oakland, CA) approved the research protocol. An online survey of 2016 graduates of eight global health-focused masters programs in the United States of America (USA) was performed between September 2016 and December 2016.

Graduates were sampled from eight public and private master’s programs in the United States, including master of public health, with a defined global health track/program; master of global health; master of global science and master of global medicine. All programs were member institutions of the Consortium of Universities for Global Health (CUGH). Descriptive statistics were conducted on the data, with graphic representation of the results.

## Results

The survey was provided to 256 individuals, and 208 (Table 1) consented to completing the study. Of the respondents, 56 were subsequently removed for not confirming graduation in 2016. Ultimately, 152 graduates from selected institutions’ 2016 classes were included in the final analysis, reflecting a 59% response rate (152/256).

**Table 1:** Demographics Information.

Question		Number (n = 152)	Percent

Degree Obtained in 2016	Master of Global Health, Global Science or Global Medicine	57	37.5%
	Master of Public Health, with a concentration in Global Health or Master of Science in Public Health	95	62.5%
Gender	Male	36	23.7%
	Female	115	75.7%
	Other/Prefer Not to Answer	1	0.6%
Race/Ethnic Background	American Indian/Alaskan Native	1	0.7%
	Hawaiian	1	0.7%
	Asian or Pacific Islander, including Indian Subcontinent	38	26.6%
	Black, not of Hispanic Origin	9	6.3%
	Hispanic Origin	14	9.8%
	White	69	48.3%
	Prefer not to disclose	11	7.7%
Currently a citizen of U.S. or a holder of a U.S. permanent resident visa	Yes	125	83.7%
	No	26	16.3%
What degrees do you hold, excluding your recent Master’s level degree?	Bachelor’s degree	112	77.8%
	Degree in Nursing	21	14.6%
	Master’s from a school of Public Health	11	7.6%
Employed and Not Employed	Employed	102	67.1%
	Not Employed	47	30.9%
	Volunteering	3	2.0%

A broad range of employment was noted among the graduates sampled. Just under one-third of respondents were not employed at the time of this survey, with no immediate prospects. Of those self-described as not employed, 18/47 (38.3%) were in another academic training program. There were 102 respondents employed at the time of this survey. The details of their jobs, including current responsibilities, are outlined in Table 1. Almost three-quarters of employed respondents were working full time (74.7%).

With regard to how jobs were found, respondents reported as follows: 58/180 (33.5%) through recommendations by friends and colleagues, 55/180 (32%) through Internet postings and 14/180 (8.1%) through university career services offices. No respondents reported finding jobs from journal postings.

**Figure 1:**
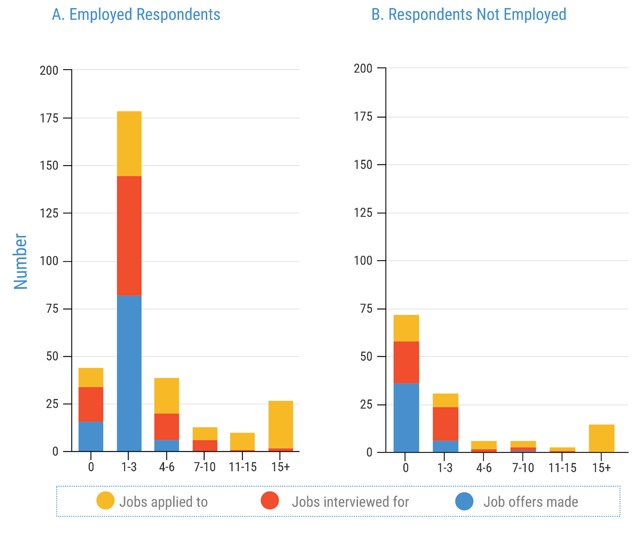
Job Applications, Interviews and Offers.

The experience of both employed and not employed respondents finding their current job is depicted in Figure 1. A majority of both number of interviews and final job offers was found in the employed group. Only 5% of respondents interviewed for more than six jobs, and 88/91 (97%) of respondents received less than four job offers. Those who were currently employed applied for, on average, between only 1 and 6 jobs, with the vast majority receiving 1 to 6 interviews and 1 to 3 job offers. Those who were not employed on average applied to more than 15 jobs, received 1to 3 interviews, and ultimately received no job offers.

The job search experiences of those who were not employed and seeking employment were very different compared to those currently engaged in an academic or training program. Out of respondents in the former group, 14/24 (58.3%) reported that they applied to greater than 15 jobs, 15/24 (62.5%) reported receiving only 1 to 3 interviews. Seventy-five percent reported receiving no job offers (Figure 1).

**Figure 2:**
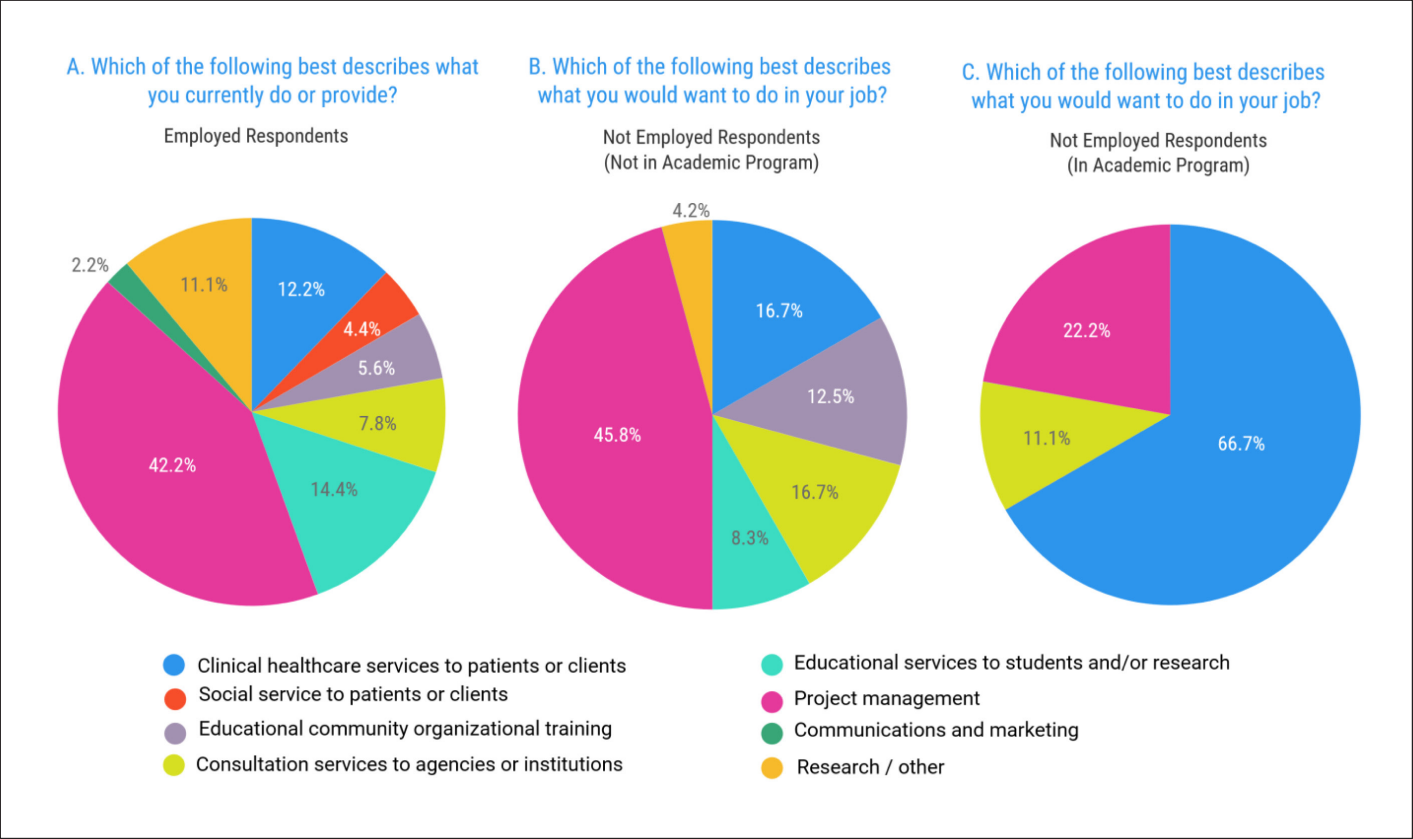
Employment Services Type.

Figure 2 depicts respondents’ job descriptions. Of employed and not employed respondents, 38/90 (42.2%) and 11/24 (45.8%), respectively, described their current and ideal jobs as project management. The next highest categories in employed respondents were educational services to students and/or research (13/90, 14.4%) and data analysis/research (10/90, 11.1%). The least common job description was communications and marketing (2/90, 2.2%).

Of those not employed and currently in an academic program, 67% stated that they would like to work in a healthcare setting. Only 4/24 (16.7%) of not employed respondents not in an educational training program stated that they would like to work clinically (Figure 2).

**Figure 3:**
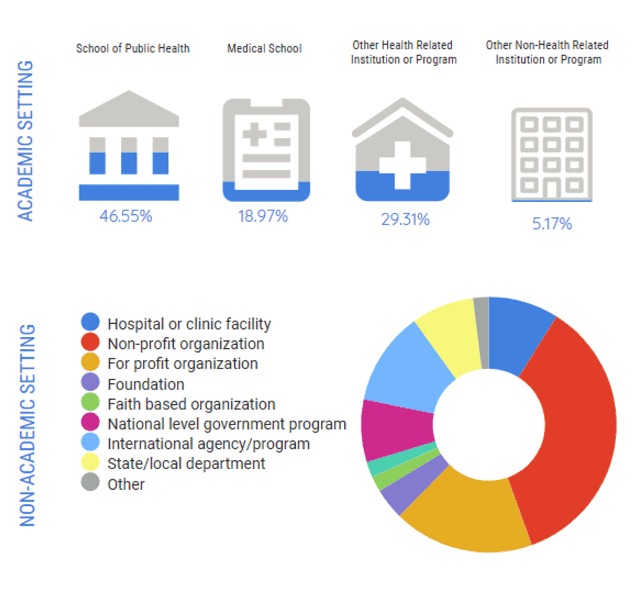
Current Academic or Non-Academic Employment Setting.

Figure 3 demonstrates that in the academic setting, schools of public health were the most common location for employment (27/58 (46.6%)) while in the non-academic setting, not-for-profit/NGOs were the most commonly cited (18/56 (32.1%)).

Categories selected least frequently by currently employed respondents included nursing schools 0/56 (0%) and faith-based organizations 1/56 (1.79%).

**Figure 4:**
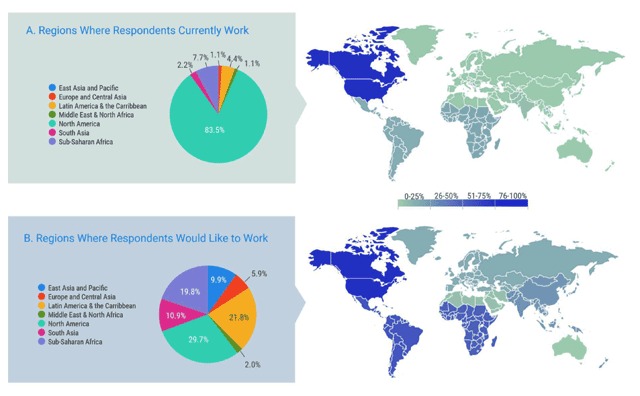
Employment Location.

Respondents were asked where their jobs are located, as well as where they would like to work. When the data are compared (Figure 4), the vast majority of respondents work in North America 76/91 (83.5%), whereas only 30/101 (29.7%) aspired to work in North America. The distribution of preferred location for work is more evenly divided amongst all World Bank analytic regions, with the most common after North America being Latin America and the Caribbean 22/101 (21.8%) and Sub-Saharan Africa 20/101 (19.8%).

**Figure 5:**
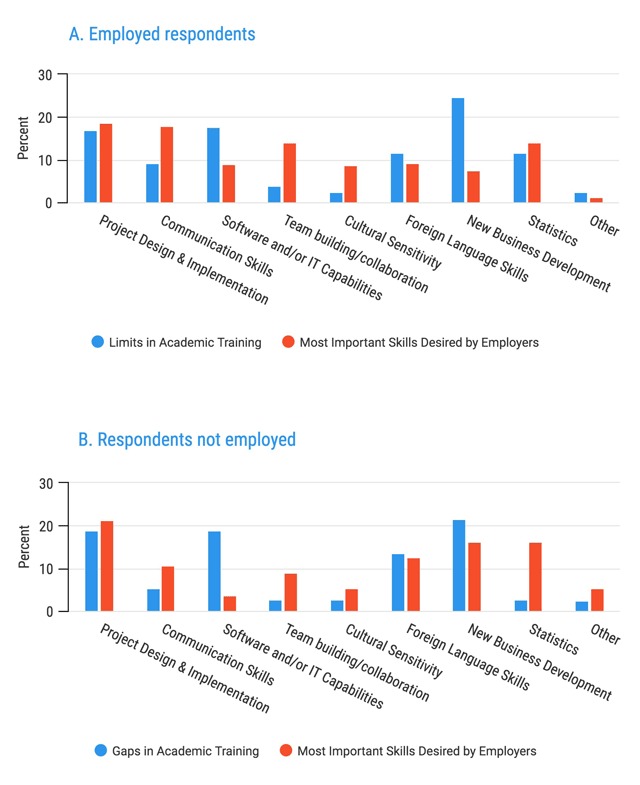
Gaps in Academic Training and Most Important Skills Desired by Employers.

Of the perceived academic training limitations noted by graduates, new business development (such as fundraising), as well as software/IT capabilities and project design implementation, were selected the most frequently (25%, 17.5% and 16% respectively, Figure 5). When compared to what respondents thought would be the most important skills to their employers, new business development was rated less important to employers than their perceived gaps in training, while project design/implementation, team building/collaboration and communication skills were higher.

**Figure 6:**
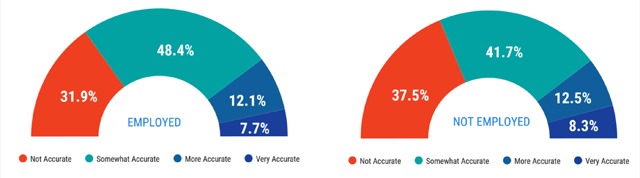
Limitations in Academic Training.

**Figure 7:**
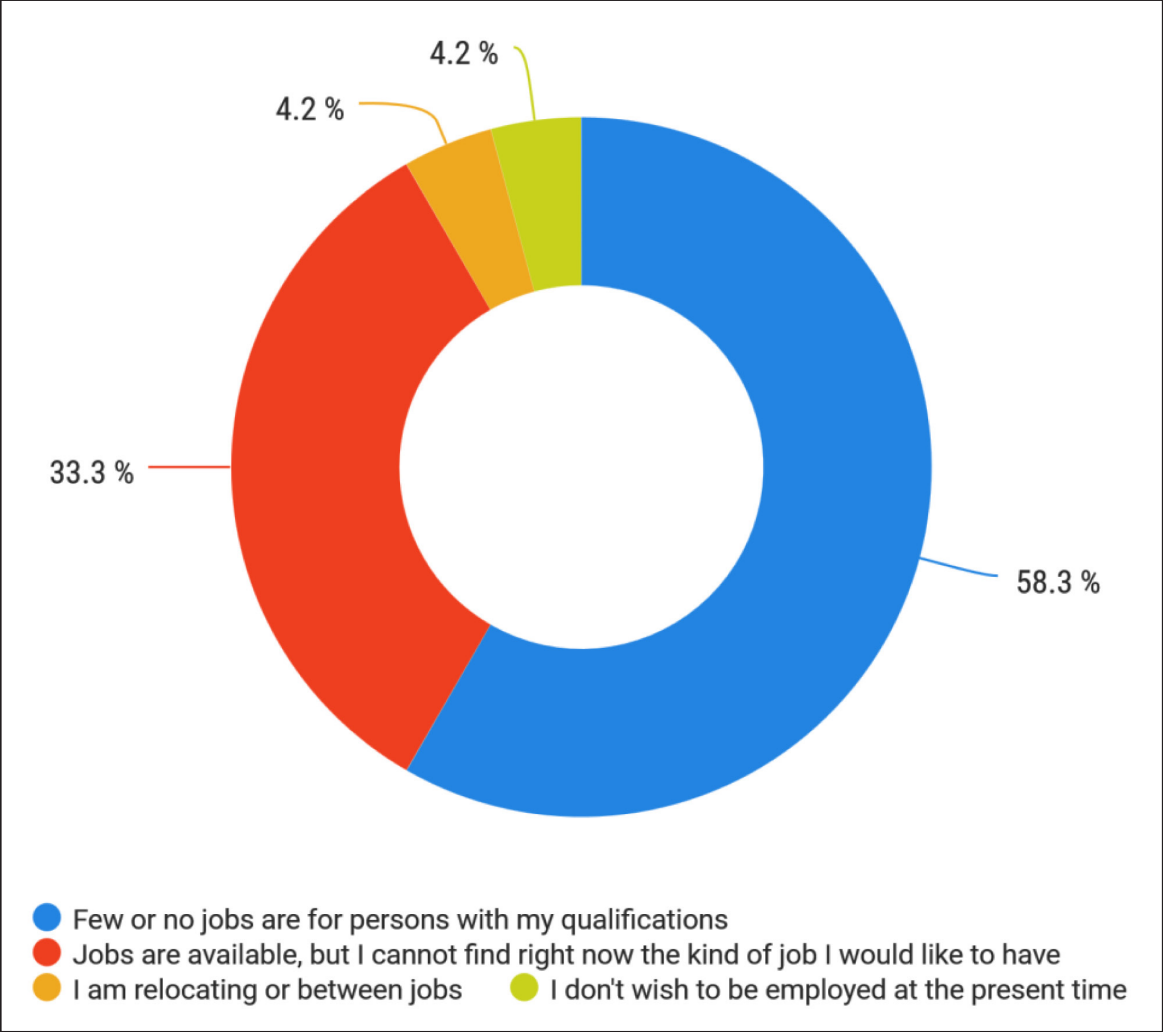
What is the Primary Reason That You Are Now Unemployed?

Out of 91 respondents, 62 (68.1%) felt that it was somewhat to very accurate that they had limitations or gaps in their academic training, as detailed above. The majority of respondents who were not employed also agreed with the statement (15/24, 62.5%) (Figure 6).

With regard to gross salary, clear trends emerged between pre-program, post-program and desired amounts. Before beginning the recent master’s-level degree, respondents on average earned $40,000 or less per year (77%). At the time of this survey, after graduation, most respondents were earning between $40,001 and $60,000 (55%). However, 41% of respondents identified that, for their current level of training and experience, they felt that they should be earning a gross yearly income of between $60,001 and $80,000.

When those not employed were asked about expected salaries upon employment, 72% of respondents who were currently in an academic training program anticipated earnings between $100,001 to >$160,000/year. Conversely, 80% of those not in an academic training program estimated a range between $40,001 and $80,000/year.

A total of 47/152 (30.9%) respondents stated that they were not employed at the time of this survey. The most common reason cited for unemployment was the lack of jobs for persons with the graduate’s qualifications (Figure 7).

## Discussion

Of the surveyed graduates of global health-focused master’s degree programs, 21% were not employed and not currently in an educational program. The U.S. unemployment rate in the same period for those with masters-level degrees was 2.4%, raising questions about the sustainability of global health-focused degree programs [19]. There were differences in the experience of searching for a job between respondents who were employed and not employed at the time of this survey.

To better understand these different experiences, we surveyed resources utilized for the job search. Professional and personal networks and internet job listings formed the backbone of most respondents’ job search processes. There was also a similar trend in use of alumni networks. Unfortunately, we did not include this question in our survey of those not employed, so comparisons between groups cannot accurately be assessed at this time.

It is apparent from the responses of graduates currently not employed that the primary challenges to finding a job are limited opportunities and gaps in skills for those that are available. This suggests a mismatch between the number of jobs available in global health and the number of global health graduates. As well, it suggests that existing global health education programs may lack curricula focused on skills sought in the global health job market. Specifically, all categories of respondents identified project design/implementation skills, statistics, new business development and software/IT services as their foremost perceived training gaps. Communication skills were added to this list in relation to perceived employer desired characteristics.

How these gaps are to be addressed by the respondents, or their employers, is beyond the scope of this study and therefore not made evident by the respondents’ selections. However, in the 2016 Employer’s Study 4, employers reported in-service training was required to address these gaps. We also believe that elements such as adaptation, on the job learning, or task sharing with colleagues and peer staff are common resulting scenarios. We also sought to identify skills that graduates perceived employers were seeking. These included program management and statistical competency, while also including communication skills, team building and collaboration, alongside cultural sensitivity and foreign language skills. These latter skills were previously noted by a survey of major employers to be lacking in job candidates with domestic experience, specifically “flexibility, adaptability, and creativity; cultural sensitivity; and cross-cultural communication skills [5].” The consistency of this identification of desired skills across both cohorts should be well noted and could help to inform further curricular intersection between the academic and employer contexts.

Interesting trends emerged amongst those who were employed, not employed but in an academic training program and not employed or in an academic program. In particular, the responses to questions about average salaries of those employed increases in roughly $20,000 increments: with an average range of $20,001 to $40,000 prior to the degree and $40,001 to $60,000 afterwards. Expected current salaries for the advanced degree were in the $60,001 to $80,000 range.

No respondents currently reported earning more than $100,000, although some did suggest they should be earning >$160,000.

This demonstrates that there is indeed a notable increase in the annual gross salary of respondents after having completed their master’s-level training in global health (a roughly 150% increase). According to national level data for the average income for those with a master’s degree of any kind, annual gross income is approximately $69,000. Figures for the national average salary ranges of those with a master of public health show that 26.8% earn between $50,001 and $75,000, and 23.8% earn between $30,001 and $50,000 [20]. This data fits with our findings but indicates a potential disconnect between what students expect going into a master’s program in global health and actual salaries. More investigation is needed to determine if perspectives should be reframed around students’ anticipated earnings and perhaps framing a realistic salary when compared to colleagues pursuing master’s level training in areas such as business administration, engineering or otherwise.

The cohort of unemployed respondents’ salary expectations matched current salaries of respondents employed at a range between $40,001 and $80,000. However, approximately 45% of respondents currently in an academic training program suggested that they would earn >$160,000. Further exploration of the data demonstrated that those respondents tended to be in MD, DDS, or veterinary medical degree programs where they are likely to work in a domestic clinical scope of practice following graduation. This anticipated salary does correlate with likely earnings following graduation from a professional degree program, such as a family medicine residency, with subsequent domestic clinical work [21].

One intended goal of this study was to better understand the barriers to employment experienced across diverse geographic and population groups. As such, multiple attempts were made to engage minority-serving institutions (MSIs) [22] at both the onset and midpoint of the survey period. While we did engage some MSI institutions, there were ultimately a limited number that fit the inclusion criteria for our study and were able to participate. Additionally, results about volunteer work were not clear. The limitation of question wording and options within Question 8 (See Appendix A) redirected three respondents who selected “volunteer” to the “employed” section. After final analysis, those respondents were placed in the “not-employed” section. When identifying current jobs, the survey did not distinguish educational research from basic science and clinical research. In the survey, “research and data analysis” were grouped within “education and research,” leading many respondents to select “other” for questions related to job functions. This was a limitation as “research” was one of the most common self-defined job titles, as evident from Question 30 (See Appendix A).

This study was a pilot of representative programs from across the continental United States. Upon final analysis, we have developed three sets of recommendations to be useful to the major audiences we believe will benefit from this article: (1) Students, (2) universities and (3) Employers. Please see Table 2 for final recommendations. In the future, studies should aim to increase the sample size, ask more questions related to job satisfaction during the job search or job hiring process and survey graduates of similar programs from around the world longitudinally.

**Table 2:** Recommendations for Students, Universities and Employers.

Major Audience	Recommendations

**Students**	When searching for graduate programs, review the core curricula, consider key competencies and potential gaps in training. Connect early with faculty, colleagues, mentors and alumni throughout the course of study to increase personal networks. Engage early in applicable work and research, paid or otherwise, to strengthen qualifications. Courses in data analysis, statistics and IT management may be useful. Consider other venues for curriculum, such as business school course in program design and implementation or an adult education course in collaboration and managing teams to enhance your skills in these key areas. Subscription to online job posting sites is recommended for improved awareness of job availability and prospective employers. Once students become active job seekers, apply to as many positions as possible that fit interests and skills. Prepare for the possibility of working in North America and earning a lower starting salary than peers with masters-level training in other disciplines (engineering, business administration, etc.).
**Universities**	Develop and maintain strong pipelines with global health employers. Seek employers’ input regarding curricular content to help match program learning outcomes with employers’ needs. Integrate training in project design and implementation, new business development, IT training, communications, team building and other skills identified by both graduates and employers in this study. Provide opportunities for internship and volunteer positions that demand project implementation. Maintain robust correspondence with program alumni to provide feedback and identify gaps in education and training. Engage students with program alumni, which will both build students’ professional networks and grant them insight into alumni experiences. Share available job postings on university web sites or through student listservs.
**Employers**	Support network building among universities, students, alumni and your own organizations. Provide recommendations for curricula, internship and volunteer opportunities in order to furnish students with non-clinical skills needed for employment. Be cognizant that job seekers are simultaneously applying to many jobs. Provide opportunities for continuing education and skill development to allow those who do obtain entry-level jobs to refine pertinent and necessary skills that may not have been acquired in education alone.
